# Antibiotics in Aquaculture Wastewater: Is It Feasible to Use a Photodegradation-Based Treatment for Their Removal?

**DOI:** 10.3390/toxics9080194

**Published:** 2021-08-21

**Authors:** Carla Patrícia Silva, Vitória Louros, Valentina Silva, Marta Otero, Diana L. D. Lima

**Affiliations:** 1 CESAM & Department of Chemistry, Campus de Santiago, University of Aveiro, 3810-193 Aveiro, Portugal; vitorialouros@ua.pt (V.L.); valentinagsilva@ua.pt (V.S.); diana.lima@ua.pt (D.L.D.L.); 2 CESAM & Department of Environment and Planning, Campus de Santiago, University of Aveiro, 3810-193 Aveiro, Portugal; marta.otero@ua.pt

**Keywords:** aquaculture industry, bacterial resistance, photolysis, photocatalysis, sustainable water treatments

## Abstract

Aquacultures are a sector facing a huge development: farmers usually applying antibiotics to treat and/or prevent diseases. Consequently, effluents from aquaculture represent a source of antibiotics for receiving waters, where they pose a potential threat due to antimicrobial resistance (AMR) induction. This has recently become a major concern and it is expectable that regulations on antibiotics’ discharge will be established in the near future. Therefore, it is urgent to develop treatments for their removal from wastewater. Among the different possibilities, photodegradation under solar radiation may be a sustainable option. Thus, this review aims at providing a survey on photolysis and photocatalysis in view of their application for the degradation of antibiotics from aquaculture wastewater. Experimental facts, factors affecting antibiotics’ removal and employed photocatalysts were hereby addressed. Moreover, gaps in this research area, as well as future challenges, were identified.

## 1. Introduction

The human population is growing at an incredible rate and that is illustrated by the numbers: from 3 billion in the early 1960s, it is expected to reach 9.7 billion by 2050. Consequently, the human requirements for animal-derived food sources have been and are expected to continue expanding due to both the increasing global population and economic growth. This holds several issues from which the worldwide demand for fish takes an important role. Fish is known to be an important source of essential micronutrients, proteins, and fatty acids and that has important and positive implications in human health [1]. Specifically, the annual global growth in fish consumption has been twice as high as the population growth over the past decades [2,3]. The claim for fish has been contributing for the expansion of the sector and the global fish aquaculture production reached its maximum at ~171 million tons in 2016 [2,3]. In the period 1990–2010, production growth rate increased 6–8%, nowadays accounting for 50% of the world’s fish that is used for food and being considered the fastest growing food sector—more than all the other sectors of animal food-production together—by the United Nations Food and Agriculture Organization (FAO) [1,2,4,5,6]. However, this has not been the only reason for the expansion of the aquaculture sector. Other factors, as wild fisheries reaching, or even exceeding, their sustainable limit, or the lack of policies that promote productivity and a high level of investment together with the improvements in technology and innovative methodologies (as the recirculating aquaculture systems (RAS)) have also accounted for the development of aquaculture industry [1].

Aquaculture may take place in freshwater, which represents ca. 60%, and in seawater or brackish water, representing ca. 32 and ca. 8%, respectively. Most of the aquaculture operations take place in the Asia-Pacific region (ca. 89%) [1]. This sector presents very important positive impacts, such as the attenuation of the pressure on natural resources, the production (and consequent consumption) of protein-rich fish, containing essential micronutrients, and also the economic development (for instance, 18.7 million people were estimated to be employed in aquaculture in 2015) [1,6].

A main issue derived from the huge growing and production of the industry is the vulnerability to noteworthy stock losses due to bacterial diseases, which solution or prevention requires the intensive use of antimicrobials [1,5]. This is closely related to the subsequent release of these compounds into the environment by the discharge of aquaculture effluents. Some of the key concerns include the potentially harmful effects of antibiotics in the environment and human health, together with the propagation of antimicrobial resistance (AMR). These effects are in contradiction with the goal of sustainable aquaculture, which should attain a balance between providing a constant source of food for human sustenance and generating no or minor harming to ecosystems. Reaching and maintaining this equilibrium is and will be a real challenge since the world aquaculture production will suffer the pressure to attain 102 million tons by 2025 in order to sustain the current consumption level [7].

It should be highlighted that most aquaculture development has arisen in the last 50 years and, thus, the concept of sustainability, in particular environmental sustainability, and the progress of practices aiming to decrease of the aquaculture environmental footprint has remarkably progressed ever since. So, even if environment was not the most demanding pressure for the industry five decades ago, it is now. This justifies the discussion raised in the present manuscript *per se*, which aims to summarize published information on photodegradation-based strategies for the removal of antibiotics in aquaculture waters. Both photolysis and photocatalysis methods will be assessed. Influencing factors and employed photocatalysts will be addressed so we will analyze if these methods are effective and if they may be implemented in aquaculture facilities. Finally, main research gaps and future needs for this field of study will be presented.

## 2. The Use of Antibiotics in Aquaculture and the Spreading of Antimicrobial Resistance (AMR)

Antibiotics, as antimicrobial agents, have substantially transformed modern medicine, saved countless lives, and prolonged life expectancy. Even though these pharmaceuticals were initially developed for human medicine, their use has rapidly broadened to animal medicine for treating bacterial diseases in livestock and aquaculture industries [3]. Moreover, their usage started to be related not only to treatment but also to prophylactic measures—for instance, in aquaculture exploitation, scenarios that boost the use of antibiotics include handling stress and related immune distress or poor hygiene, that typically lead to disease outbreaks [3,8]. The natural consequence following the discovery of both disease fighting/prevention and growth promoting potential of antibiotics was fish farmers and livestock producers starting to use such substances widely [6]. As a result, to meet both the advantages in terms of health and the high market-driven demands, the use of antibiotics has become an important part of the current industrial-scale fish production [3,9]. The most common way for the medication of fish with antibiotics is by mixing them with specially formulated feed. However, nearly 80% of the antimicrobials used in aquaculture end up uneaten (when used as medicated feeds) and/or unabsorbed [3]. On the other hand, even if ingested, fish do not effectively metabolize completely the pharmaceuticals, excreting them into the water in a percentage that has been estimated to be around 75% [5]. Therefore, antibiotics may easily enter the aquatic environments surrounding aquaculture facilities [3]. Additionally, in the specific case of RAS, a main disadvantage is that the recirculation increases the animals’ exposure to antibiotics. However, they are advantageous over conventional aquaculture systems, since RAS are closed systems that enable up to 90–99% of the water to be recycled, vastly reducing water consumption—100 times lower than in traditional flow through systems [10]—and improve waste management, reducing potential negative impacts on the environment and ecosystems [1]. In fact, sanitary barriers and measures used in terrestrial food animal production are more challenging to create in aquaculture [11].

There is scientific evidence about the fact that the use of antibiotics in food-producing animals can lead to antibiotic resistance, which can then be transmitted to the general population, causing treatment-resistant infections. Non-pathogenic bacteria can be also affected by the intensive use of antibiotics, being that their resistance genes can be transferred to disease-causing bacteria, resulting in antibiotic-resistant infections for humans [6]. Therefore, both animals and animal waste are a potential reservoir of multi-resistant genes that can be transmitted directly or indirectly to humans through contact and food consumption [3,9]. There are two main mechanisms for AMR: (i) an inherent or intrinsic resistance, which occurs when a bacterial species is not susceptible to the antibacterial agent, due to the inability of the latter to reach the target site inside the cell; or (ii) an acquired resistance, when the bacterial species is susceptible to the antibiotic, but some strains are resistant and proliferate under the selective pressure induced by the use of that agent [9]. Some regulatory agencies have started to implement measures to combat AMR. One example is the Global Action Plan on AMR launched by the World Health Organization (WHO) in 2015, which provides a framework for developing key actions to be taken within 5–10 years [12]. Europe has also taken several actions, as the European Commission Action Plan Against AMR or the European Union (EU) Directives that have settled watch lists that include antibiotics for EU-wide monitoring.

However, not only the induction of AMR is a concern when thinking about the presence of antibiotics in the environment. Antibiotic residues may accumulate in sediments, drive modifications in microbial communities, enforce toxic effects in non-target species, and modify phyto and zooplankton diversity. In consequence, the food chain may change, changing every level within the ecosystem [8]. So, the consequences of the use of antibiotics are wide. That is why antibiotics used in aquaculture must be approved by regulatory agencies, which may set rules for their use, including in what respects transportation, dose rates, withdrawal times, and tolerances [5]. Nevertheless, and despite some regulatory efforts, antibiotics are still widely administered in aquaculture around the world [3].

From all the above, it is clear that, in order to ensure the sustainable development of the aquaculture industry, and along the reduction and optimization of the use of antibiotics, it is crucial to evaluate and understand the persistence pattern of these pollutants. And, in addition, it is of paramount importance to develop strategies to treat aquaculture effluents for the removal of antibiotics before their release into the environment.

## 3. Photolysis of Aquaculture Antibiotics: Influencing Factors

It is well known by now that organic pollutants, as antibiotics, represent a threat to the aquatic environment, with deleterious effects such as acute/chronic toxicity to aquatic organisms, accumulation in ecosystems, loss of biodiversity, and, ultimately, risks to human health [10].

To correctly evaluate the real ecological impact of pollutants, it is essential to take into consideration their environmental fate and persistence. Once present in water, antibiotics may be subjected to various transformation and removal processes, namely, biodegradation, sorption to colloids and sediments, and photodegradation. Sorption has been thought as the key process regulating the mobility/transport of antibiotics but that does not underestimate the importance of photodegradation, which is also considered as one of the most important mechanisms affecting the environmental persistence of pollutants, especially in surface waters [13,14]. The photochemical fate of antibiotics in natural aquatic environments and in pure water may differ significantly, owing to the presence of naturally occurring radiation absorbers, quenchers or sensitizers. The lifetime of these contaminants may thus depend on the compound in question, the degradation pathway and differences in chemical composition of natural water, that invariably affect the photochemical function [15,16].

**Figure 1:**
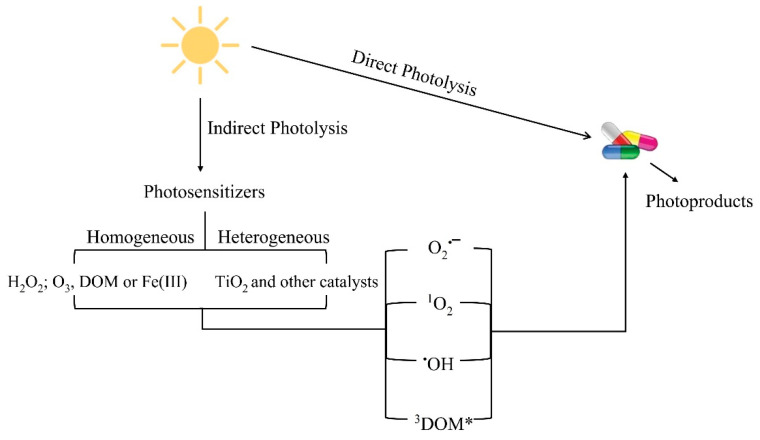
Representation of photolysis pathways of organic compounds in aquatic matrices.

Photodegradation may happen in different ways and may be sensitized or hampered by different factors (Figure 1).

Direct photolysis happens when chromophoric groups can absorb light at wavelengths present in sunlight (λ > 290 nm) [17], involving absorption of photons by the pollutant itself, which makes it capable of inducing a chemical transformation [13,18]. Consequently, the rate of direct photodegradation is a function between different variables: the available light intensity, the pollutant’s capacity to absorb that light and the efficiency of the conversion of the absorbed light into photochemical reactions [19]. On the other hand, energy can be transferred to dissolved oxygen to form reactive oxygen species (ROS; hydroxyl radicals (^•^OH), peroxyl radicals (ROO^•^), hydroperoxyl and superoxide radicals (HO_2_^−^/O_2_^−^), and singlet oxygen (^1^O_2_)), which subsequently cause the self-sensitized photooxidation of the pollutant. Indirect photolysis happens when other substance, named as photosensitizer, indirectly induces the chemical transformation. Such a substance, after absorbing the solar radiation, reaches an excited state and then generates free radicals (ROS or non-ROS transients as reactive halogen species (RHS)) [18,20], which cause the phototransformation of the contaminant. Photosensitizers may be naturally present in water matrices, with chromophoric dissolved organic matter (CDOM) (DOM that absorbs light) and different Fe(III) polycarboxylates being some of the most important types [18]. As for the latter, Fe(III)-carboxylate complexes (with oxalic, citric, lactic and pyruvic acids) present great ability to generate ROS (mainly ^•^OH radicals). In the photochemical cycle, photogenerated Fe(II) ions and organic radicals react with the dissolved oxygen which leads to the restoration of Fe(III), the oxidation of organic compounds and the formation of ROS, which can effectively oxidize contaminants as pharmaceuticals [21,22,23]. As for the effect of DOM, and since its study has a higher expression in literature regarding the photolysis of aquaculture antibiotics, it will be addressed in more detail in Section 3.3.

Table 1 displays important data reported in literature on the degradation of aquaculture antibiotics by photolysis. As evidenced by published results, the photolysis rate may be affected by different factors, which will be addressed in the next sub-sections.

### 3.1. Type of Light

Since photodegradation is directly related to photon absorption either by the compound itself or by photosensitizers, the type of light used in these processes is extremely important. As it may be seen in Table 1, most published works on the photodegradation of aquaculture antibiotics use natural or simulated sunlight, which is supported by the sustainability of using solar irradiation for water treatment. Scarcer are works dealing with UV irradiation, even when its use for water disinfection in aquaculture is extended [24]. Yet, some authors have compared the influence of the type of light on the photolysis of aquaculture antibiotics. Lin et al. (2010) [25] studied the photodegradation of enrofloxacin (ENR) and ciprofloxacin (CIP), both fluoroquinolone antibiotics, under ultraviolet (UV) and fluorescence light, when present in overlying water collected from an eel pond. UV and fluorescence light both presented similar effects on the degradation of ENR, while CIP degradation was faster by the action of UV rather than fluorescence light treatment. Authors concluded that their degradation under dark conditions was very much delayed, confirming that, for both antibiotics, degradation was mostly related to the action of light, with biodegradation playing just a minor role. As for CIP, natural sunlight irradiation was already proved to be efficient to substantially photodegrade the antibiotic with the process not being hindered by the presence of non-target compounds [26]. Ge et al. (2009) [24] also studied the effect of different types of light, in this case on the photodegradation of two phenicols, namely thiamphenicol (THI) and florfenicol (FLO). Authors found that under UV-vis irradiation (λ > 200 nm) and under real or simulated sunlight (λ > 290 nm), the behavior was different depending on the matrices tested. As for the UV-Vis radiation, phenicols degraded more rapidly in seawater, followed by pure and freshwater, whilst for solar radiation (either real or simulated) they only photodegraded in freshwater. Therefore, photodegradation kinetics of THI and FLO were shown to be dependent on the light type in conjugation with the type of water constituents.

**Table 1:** Photolysis of antibiotics used in aquaculture.

Reference	Antibiotics	Irradiation	Relevant Conditions	Conclusions
[25]	ENR; CIP	UV and	ENR and CIP: 500 mg L^−1^	CIP degraded faster with UV than with fluorescence light treatment;
		Fluorescence	Overlying water from an eel pond	no such difference was found for ENR
[15]	TRIM;	Sunlight	River water related	*t*_1/2_ ranged from <1 to 44 days, depending on the availability of sunlight
	ENR; SDZ		to intensive aquaculture	TRIM was not susceptible to photodegradation, but ENR and SDZ were
[27]	OMP	Simulated sunlight (450 W Xe lamp)	OMP: 36.8 mg L^−1^ or 368 mg L^−1^	Photodegradation was higher in real samples in comparison with ultrapure water
			Water from aquaculture	DOM increased photodegradation in relation to direct photodegradation
			facilities and nearby stream	Indirect photodegradation pathway occurred by ^·^OH radicals, O_2_ attack, and reaction with ^3^DOM*
[28]	OXA; FLU	UV simulated	OXA and FLU: 5000 mg L^−1^	Light played a major role in the degradation of both OXA and FLU
		natural light	Water from aquaculture ponds	Temperature did not affect the photodegradation of both OXA and FLU
[29]	CIP	Simulated sunlight	CIP: 1657 mg L^−1^	Due to the Cu(II) complexation, photodegradation, photolytic pathways and
		(1000 W Xe lamp)	Ultrapure water	product distribution were altered
[30]	OTC; OXA; FLU; FLO	Wavelength range	OTC, OXA, FLU, FLO:	FLO was not degraded by photolysis
		300–800 nm	1000 mg L^−1^	Photolysis was responsible for about 70% of the OTC degradation in both
			Deionised water, fresh	fresh and seawater and 10% of the OXA and FLU degradation in seawater
			and seawater	Water pH played an important role in OTC photolysis
[31]	OTC	Simulated sunlight	OTC: 4 mg L^−1^	*t*_1/2_ predicted for OTC ranged between 21 and 25 min
		(1500 W Xe lamp)	Water from aquaculture	High pH and presence of sea salts increased the OTC photodegradation
			facilities	rate in comparison with deionised water
[32]	OTC	Simulated sunlight	OTC: 4 mg L^−1^	Mg^2+^ inhibited the formation of some OTC photoproducts observed in the
		(1500 W Xe lamp)	Water from aquaculture	presence of Ca^2+^
			facilities	Two new OTC photoproducts were formed in the presence of Mg^2+^
[33]	NOR	Simulated sunlight	NOR: 1597 mg L^−1^	DOM influenced the photodegradation of NOR different species
		(350 W Xe lamp)	Ultrapure water	Influence was related with DOM concentration and type of NOR species
[34]	THI; FLO	UV-vis: 300 W	THI and FLO: 2–30 mg L^−1^	The photolysis kinetics in pure water was influenced by the initial
		high-pressure Hg	Ultrapure water	concentration of antibiotics
		lamp (λ > 200 nm)		Twelve intermediates were formed
[24]	THI; FLO	UV-vis: 300 W	THI and FLO: 10–400 mg L^−1^ Ultrapure, sea and fresh water	Under UV-vis irradiation, antibiotics photodegraded the fastest in seawater, followed by ultrapure water and freshwater
		high-pressure Hg		
		lamp (λ > 200 nm)		Under solar (natural or simulated) sunlight, photodegradation occurred just in freshwater
		Sunlight: λ > 290 nm		
		Simulated sunlight:		
		1000 W Xe lamp		
[35]	OXA	Simulated sunlight (1500 W Xe lamp)	OXA: 100 and 250 mg L^−1^ Fresh and brackish water	Photodegradation rate constant decreased from 0.70 ± 0.02 h^−1^ in ultrapure water to 0.42 ± 0.01 h^−1^ in freshwater and to 0.172 ± 0.003 h^−1^ in brackish water ^1^O_2_ played an important role in OXA photodegradation process

CIP—Ciprofloxacin; ENR—Enrofloxacin; FLO—Florfenicol; FLU—Flumequine; NOR—Norfloxacin; OMP—Ormetoprim; OTC—Oxytetracycline; OXA—Oxolinic acid; SDZ—Sulfadiazine; THI—Thiamphenicol; TRIM—Trimethoprim.

### 3.2. Presence of Ions

Generally, natural waters and wastewaters are complex matrices, which commonly contain a diversity of ions. These ions can react with radicals, thus affecting organic compounds’ degradation. For instance, it is referred in literature that some ions, such as chloride and bromide, can scavenge the ^•^OH, decreasing photodegradation [36]. On the other hand, concomitant pollution between antibiotics and metals may happen, particularly in natural waters nearby aquaculture zones where both (antibiotics and metals) are used as feed additives. In this case, metal complexes may be formed altering the physicochemical properties and chemical reactivity of antibiotics, which in turn may change the fate and toxicity of those compounds [29]. Based on this assumption, Wei et al. (2015) [29] explored the role of the concomitant presence of metal ions on the photodegradation of CIP and found that copper ions’ complexation can alter the light absorption, the photodegradation pathways, and the ^1^O_2_ photogeneration, inhibiting the photodegradation [29].

In the case of oxytetracycline (OTC), solar photodegradation was observed to be faster in marine aquaculture’s water than in deionized water by Leal et al. (2016) [31]. In a subsequent work ([32]) the authors studied the effect of calcium and magnesium ions on the OTC photodegradation kinetics. Compared with the previously determined photodegradation kinetic rate constant (*k* = 0.082 ± 0.001 min^−1^) [31], an increase of ~21% (*k* = 0.099 ± 0.004 min^−1^) was obtained by the addition of calcium (500 mg L^−1^) to the aquaculture’s water [32]. Therefore, authors concluded that OTC photodegradation was driven by the calcium ion, which formed complexes with higher photolysis quantum yields. However, in presence of magnesium ions, inhibition of the sensitizing effect of calcium was verified [32]. Differently from calcium, which affected OTC kinetics, magnesium induced the formation of by-products not detected in its absence. Additionally, in this work, a lower quantum yield of the direct photolysis of the complexes with magnesium is proposed. Similar to the calcium ions, also the iron ions are known to have strong affinity with tetracycline antibiotics, establishing complexes with OTC. In the photochemical process, complexation of Fe(II) with OTC led to accelerated oxidation of Fe(II) and simultaneously promoted the degradation of OTC [37].

As for phenicols, Ge et al. (2009) [24] observed that the photodegradation of THI and FLO under UV-vis irradiation was faster in seawater followed by pure water and freshwater. Authors ascribed this acceleration to the photosensitized generation of ^1^O_2_ that occurred in the presence of chloride ions. 

In the specific case of metal ions, different complexation effects have been observed on the photochemical behavior of antibiotics and their dissociation species. The study of such effects is particularly necessary in the case of antibiotics in aquaculture water due to the varied composition of aqueous matrices. 

### 3.3. Presence of Dissolved Organic Matter (DOM)

As already referred, CDOM is the colored fraction of DOM able to absorb radiation. Redox reactions are the dominant reaction type between organic substrates and the triplet state of CDOM (^3^CDOM*), with ^3^CDOM* primarily acting as the oxidant [38]. Even though chemical forms of CDOM are poorly understood, it is well known that their color comes predominantly from humic substances (HS) [39], which are main constituents of CDOM. When absorbing photons in the UV and the visible region of the solar spectrum up to 500 nm it makes a variety of photochemical processes feasible [40]. Sunlight irradiation of natural water causes the transition of HS to excited states by the absorption of solar radiation [40], as follows: HS → ^1^HS* → ^3^HS*. HS have been considered as important natural photosensitizers, able to notoriously increase the photodegradation rate of different pollutants [41,42,43,44]. However, the role of HS is singular since they can also retard phototransformation [36,44], which possibly occurs by sunlight screening. In this case, HS act as inner filters, reducing the quantity of photons available for the photoreactions of the pollutant [20,45,46]. In addition, it has been proven that HS act themselves as quenchers originating contaminants’ back-reductions to the parent compound [20]. 

The effect of DOM in general, and HS in particular, on the photodegradation of aquaculture antibiotics is therefore a balance between the two opposite effects, namely by quenching radicals and inner filter effect (and thus slowing down pollutants’ photodegradation) or by acting as photosensitizer (so increasing the photodegradation rate). Guerard and Chin (2012) [27] studied the photodegradation of the antibiotic ormetropim (OMP) in real samples from a catfish aquaculture and a nearby stream under simulated sunlight radiation. Authors compared obtained results with those in ultrapure water and in presence of DOM. Photodegradation vastly increased in both samples in comparison with ultrapure water: half-life times (*t*_1/2_) decreased from 68.6 h in ultrapure water to 10.3 and 28.3 h in aquaculture sample and the nearby stream sample, respectively. Such an increase was consistent with that observed for OMP photodegradation in presence of DOM, specifically the fulvic acids (FA) fraction. In Liang et al. (2015) [33], the effect of DOM of three different types (Suwannee River FA, Elliott soil Humic Acids (HA) and Leonardite HA) was assessed for the photolysis of the fluoroquinolone norfloxacin (NOR). These authors found that, for the three types, DOM influence was different depending on its concentration and on the different species of NOR. In the case of zwitterionic and anionic species of NOR, DOM had a retarding effect in their photodegradation. Meanwhile, for cationic NOR, the effect varied with the DOM concentration, and, within the studied range, it was favored at the lower DOM concentrations (from 2 to 5 mg L^−1^, which are typical values in seawater), but retarded at the highest tested concentration (10 mg L^−1^, which resembles estuarine water). Photodegradation rate of the zwitterionic and anionic species being almost seven times faster than that of the cationic species was explained by the differences in the species’ quantum yields. 

Li et al. (2014) [34], as well as Ge et al. (2009) [24] in an earlier work, highlighted the photosensitizing effect of organic matter, concluding that photodegradation of phenicols under solar radiation is sensitized by HA. Not only the effect of HA, but also of other DOM fractions (FA and XAD-4) on the photodegradation of oxalinic acid (OXA) were investigated by Louros et al. (2020) [35]. Authors observed an inverse correlation between the HS aromaticity (HA is the most hydrophobic fraction, followed by FA and XAD-4) and their photosensitizing on OXA. Therefore, HA was the fraction presenting the lowest photosensitizing effect, which was related to their higher aromaticity and tendency to absorb light, so having a higher inner filter effect [35]. Although the presence of DOM has mostly been related with effects on the photodegradation of aquaculture antibiotics (either decreasing or increasing the photodegradation rate), the absence of effects has also been reported (e.g., [30]).

### 3.4. Relevance of Radical Oxygen Species (ROS)

In what respects the relevance of oxygen species on the photodegradation, reports vary. Guerard and Chin (2012) [27] concluded that for OMP the mechanism of indirect photodegradation occurred through ^•^OH radicals, ^1^O_2_ attack, and reaction with the triplet excited state of DOM (^3^DOM*). As for NOR, Liang et al. (2015) [33] pointed to ^•^OH and ^1^O_2_ as the ROS involved in direct photolysis and self-sensitized photolysis. Regarding CIP, a broader conclusion was attained by Wei et al. (2015) [29], who stated that dissolved oxygen should be playing an important role in its photodegradation, since rates determined in nitrogen-saturated solutions were higher than those in aerated solutions [29]. In the case of phenicols, Ge et al. (2009) [24] pointed also to the role of ^1^O_2_ (rather than of ^•^OH or O_2_^•−^). Authors also inferred that the photoreactions of the two phenicols involved the self-sensitized photo-oxidation process through ^1^O_2_. As for six different fluoroquinolones (CIP, danofloxacin (DAN), levofloxacin (LEV), sarafloxacin (SAR), difloxacin (DIF) and ENR), later on, the same authors [47], determined a negative effect of *C*_0_ on the kinetics that was attributed to self-sensitization via ROS, such as ^•^OH, via H_2_O and dissolved O_2_ serving as quenchers of triplet excited fluoroquinolones and being transformed into ^•^OH, which may strongly decrease/eliminate the direct photolysis [47]. The importance of ROS was also highlighted by Cacciari et al. (2016) [48] for the case of vancomycin (VCM), and by Ge et al. (2018) [49] for the photodegradation of sulfonamides (SAs). In the study by Ge et al. (2018) [49], the absence of ROS induced a photodegradation lower than 2%. Additionally, the kinetics of SAs towards ^•^OH and ^1^O_2_ radicals were found to be dependent on pH (due to the different reactivities of the different protonated states) [49]. ^1^O_2_ radicals were also considered relevant for the photodegradation of a SA by Hao et al. (2019) [50] as well as for the photodegradation of TC by Chen et al. (2008) [51]. In this case [51], the radical generation rate decreased with increasing pH. In the case of OXA, ^1^O_2_ was also the species pointed to be responsible for the photodegradation [35]. In the presence of a ^1^O_2_ scavenger, authors verified a marked delay of OXA photodegradation, suggesting the important role of ^1^O_2_ in the process. Contribution of ROS on the photodegradation process has been admitted as essential, and it is clear that information on the species that are involved on the mechanisms is still an important topic of research.

### 3.5. Type of Matrix: Ultrapure Water, Aquaculture Representative Waters or Real Aquaculture Waters

At an initial stage, it is important to assess antibiotics photodegradation in ultrapure water. Although, it is essential to study the behavior in environmental matrices, including aquaculture water, so to find out matrix related effects (if any) in antibiotics photodegradation behavior. Guerard and Chin (2012) [27] compared the simulated sunlight photodegradation of the antibiotic OMP in samples from a catfish aquaculture and a nearby stream, with that in ultrapure water. Photodegradation vastly increased in both environmental samples in comparison with ultrapure water: *t*_1/2_ decreased from 68.6 h in ultrapure water to 28.3 h in the nearby stream sample and 10.3 h in the aquaculture sample, which was a good indication of the potential of photodegradation to treat contaminated aquaculture waters.

Lai and Lin (2009) [28] studied the photodegradation of OXA and flumequine (FLU), both belonging to the quinolones class, which are frequently used in both fresh and saltwater aquacultures due to the good relation between clinical effects and dosage. Authors assessed the effect of irradiation, for which carried out experiments under an average intensity of illumination of 1.18 MJ cm^−2^ day^−1^, which considered similar to that at the surface of an aquaculture pond, and both microbial degradation and possible abiotic degradation, by experiments under dark conditions. They concluded that simulated natural light had a significant effect on the photodegradation of both antibiotics while degradation was negligible in the dark. Under irradiation, the *t*_1/2_ observed for the two types of ponds studied (eel (freshwater matrix) and shrimp (saltwater matrix)) were quite long and did not differ much from each other: for OXA, *t*_1/2_ varied from 2.3 (eel pond) to 4.8 days (shrimp pond), while for FLU, *t*_1/2_ was between 1.9 (shrimp pond) and 2.3 days (eel pond). Therefore, OXA degradation was faster in the eel than in the shrimp pond water, while no significant differences in FLU degradation were found between the two types of aquaculture. Salinity was a determinant factor for differences in OXA degradation since the two samples had discrepant values, while the rest of the parameters (pH, dissolved oxygen, organic matter content) were similar. 

Some published studies on the photodegradation of aquaculture antibiotics do not include results in real aquaculture waters, but in other relevant related matrices, also contributing to improve the knowledge on the persistence of these compounds. Among these studies is the work by Pouliquen et al. (2007) [30] that assessed the photodegradation of OXA, OTC, FLU, and FLO in three different matrices—deionized water, freshwater, and seawater—the latter two being considered as representative of the respective types of aquaculture. Authors carried out 14-day experiments under exposure to darkness or light (1400 lux). The type of water had a high impact on the antibiotic photodegradation: FLO was very stable to photodegradation; OXA and FLU were sensitive to photolysis just in deionized water and seawater; while OTC, which was the most sensitive to photolysis, photodegraded more remarkably in fresh and seawater than in deionized water. Also, for OTC, results by Leal et al. (2016) [31] allowed to conclude that its photodegradation rate in marine aquaculture samples was ca. 3.9 times higher in comparison with that observed in deionized water—determined by the same authors in a previous work ([52]). Differently, in the case of OXA, Louros et al. (2020) [35] found a decrease in the photodegradation rate constant from 0.70 ± 0.02 h^−1^ in ultrapure water to 0.42 ± 0.01 h^−1^ in freshwater and 0.172 ± 0.003 h^−1^ in brackish water. Therefore, authors concluded that the persistence of this antibiotic is longer in environmental matrices, especially those with high salinity, than in ultrapure water. Fresh and marine waters were used as matrices for the study of THI and FLO photodegradation by Ge at al. (2009) [24]. Authors highlighted that these waters are important immediate points of entry of aquaculture antibiotics into the environment. Under solar radiation (λ > 290 nm), photodegradation rates were faster in freshwater than in seawater, which was highlighted to affect the fate of the studied phenicols and their movement from freshwater to estuaries, where lower degradation rates may enhance accumulation and associated risks. Both THI and FLO did not photodegrade in ultrapure water under solar radiation, but they did when exposed to UV-vis irradiation (λ > 200 nm). Therefore, Ge et al. (2009) [24] concluded that UV-vis could be used to remediate these phenicol antibiotics in aquaculture effluents (being particularly suitable for saline aquacultures), given that this type of irradiation is already widely used for disinfection of aquaculture systems.

### 3.6. Temperature

Lai and Lin (2009) [28] studied the photodegradation of OXA and FLU, stating that the degradation of both antibiotics was insensitive to temperature in the range 10–55 °C. Further, authors confirmed that temperature discrepancies during aquaculture processes do not have significant effects on the degradation of these antibiotics. As for OTC, OXA, FLU and FLO, Pouliquen et al. (2007) [30] stated that, within 5–20 °C, temperature had no effect neither on their hydrolysis or their photolysis in deionized water. Differently, in a study by Li et al. (2018) [53], discrepancies in the photodegradation rates (between 0.004 and 0.036 h^−1^) of the SA sulfamethoxazole (SMX) were attributed to the different light intensities and temperatures in the experiments separately carried out at different time. However, these authors did not assess the effect of temperature on SMX photodegradation. Overall, the effect of temperature on photodegradation of aquaculture antibiotics is not a widely studied topic. In any case, according to published results, temperature does not seem to have an important effect on their photolysis.

### 3.7. pH

**Figure 2:**
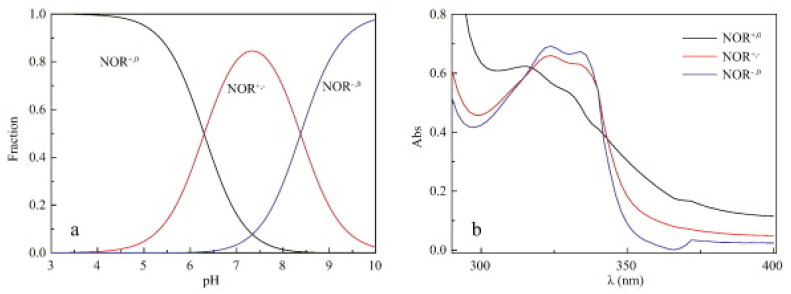
Fraction distribution (**a**) and UV-vis absorbance spectra (**b**) of the three dissociated species of norfloxacin (NOR). Reprinted from Liang et al. [33] Copyright (2015), with permission from Elsevier.

The pH may affect the properties of organic compounds in solution by altering their protonation state. A main issue is that the different protonation states may present different absorption spectra, as evidenced by Liang et al. (2015) [33] for NOR (Figure 2). 

This is also the case of OTC, which structure is altered by pH variations that, in turn, modify the absorption spectrum of OTC and therefore affect the photodegradation efficiency. Leal et al. (2016) [31] assessed the effect of pH on OTC photodegradation by comparing photodegradation results obtained in a previous work at pH 4.6 (in deionized water ([52])) with results at buffered pH 7.3 (in phosphate buffer, resembling typical values in aquaculture waters), and in aquaculture’s waters from two different companies (7 < pH < 7.5). Based on the respective kinetic constants, authors stated that pH could be responsible for 43–55% of photodegradation rate increase in aquaculture’s waters. This was related with the fact that, at pH 4.6, OTC is mostly neutral while at 7 < pH < 8, it varies from neutral to negative states. Therefore, pH has not a direct but an indirect effect on the photolysis of aquaculture antibiotics, which protonation state and light absorption are dependent on the pH of the aqueous medium.

### 3.8. Salinity

Salinity is another factor that may be involved in the photodegradation process. For the case of OTC, the presence of salinity, studied by using OTC in a phosphate buffer solution with sea salts, enhanced 1.7 times the photodegradation rate when compared with the solution of OTC in phosphate buffer alone ([31]). In this case, though, it was concluded that the improvement of the photodegradation was due to a conjugation between a higher pH and a higher ionic strength. This conjugation may affect the speciation of OTC favoring the negatively charged form of the molecule. In the case of OXA, the influence of salinity on photodegradation was assessed by Louros et al. (2020) [35], who studied two different sources of salinity preparing the solution of OXA in 21‰ NaCl solution and in a 21‰ synthetic sea salts solution. A decrease in OXA photodegradation was observed for both salty solutions when compared with ultrapure water. However, such a decrease was especially abrupt in the synthetic sea salts solution with the t_1/2_ increasing from 0.99 ± 0.04 h (ultrapure water) to 4.25 ± 0.04 h (synthetic sea salts). The decrease in the photodegradation rate may be attributed to OXA stabilization due to chelate formation between the compound and the cations, which was probably favored in the sea salts solution due to its more complex composition, containing ions such as calcium, magnesium, and carbonates [35]. 

### 3.9. Initial Concentration of Antibiotic

Li et al. (2014) [34] examined whether the initial concentration of the phenicol antibiotics had an impact on their photolytic kinetics. It was concluded that the rate constant was affected by the initial concentration of the antibiotics, since higher rates were observed for lower initial concentrations [34]. The same tendency was also observed by Louros et al. (2020) [35] for OXA, with photodegradation occurring faster for 100 than for 250 μg L^−1^ OXA (*k* = 1.24 ± 0.02 and 0.70 ± 0.02 h^−1^, respectively) [35]. In fact, it was already known that the photolysis rate may decrease due to photon limitations that may happen at higher initial contaminant concentrations [14]. This phenomenon has been observed by several authors for different pollutants: clofibric acid [54], carbamazepine [54], 17*α*-ethynylestradiol [55,56], 17*β*-estradiol [55,57], estrone [57], estriol [46], and nonylphenol [58].

## 4. Photocatalytic Removal of Aquaculture Antibiotics from Water: Types of Photocatalysts

Even though photolysis could be a valid option for the removal of pharmaceuticals from water, sometimes a faster degradation may be needed. Photocatalysis has been successfully applied for the removal of pharmaceuticals, including antibiotics [59,60,61,62,63]. In fact, it presents unique characteristics that allow for some advantages like easily-reachable reaction conditions, the use of the air oxygen to produce a powerful oxidant, and the use of solar radiation as energy source [64]. Additionally, it has been shown that photocatalysis allows for the mineralization of antibiotics to CO_2_, water and inorganic compounds, or their partial degradation to less harmful and/or more biodegradable compounds [64,65].

**Figure 3:**
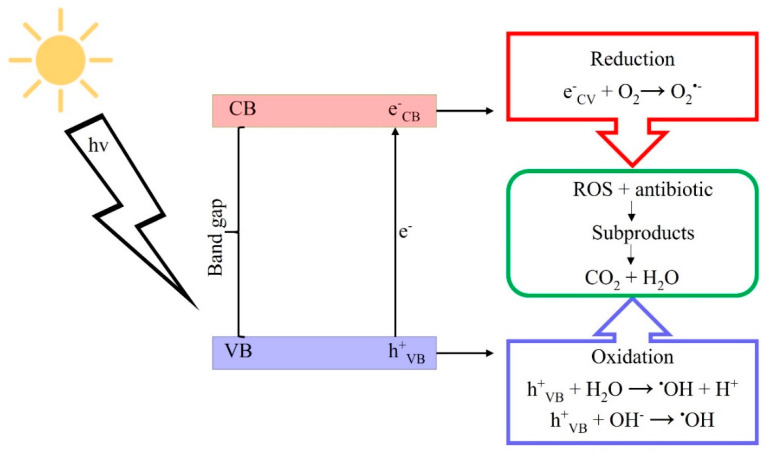
Representation of photocatalytic mechanism of heterogeneous photocatalysis conducted by semiconductors.

Photocatalysis, represented in Figure 3, is a phenomenon in which materials—the so-called photocatalysts—change a chemical reaction rate on exposure to light. In essence, photocatalysts are all semiconductors, so their irradiation results in electron-hole pair generation, providing energy equal to, or greater than, the bandgap [66,67,68]. The photo-generated electrons can react with electron acceptors, such as oxygen and/or metal cations (photo-reduction), while the photo-generated holes may react with electron donors such as organic materials (photo-oxidation) [68]. 

The efficiency of a photocatalyst depends on different aspects, such as solution pH and temperature, photocatalyst-pollutant contact time, photocatalyst dose, and irradiation time [69]. Metal oxide semiconductors have been used as photocatalysts for the degradation of pharmaceuticals, including antibiotics, either pure [61,70] or doped with other materials [71,72,73]. Photocatalysis’ reactions may be categorized into homogeneous photocatalysis (when the semiconductor and the reactant are in the same phase) and heterogeneous photocatalysis (when the semiconductor and the reactant are in different phases). Heterogeneous photocatalysis has been widely used for water cleaning, its major advantage relying on the capacity to be performed under environmental conditions [66,67].

Titanium dioxide (TiO_2_) is one of the most widely used semiconductors in photocatalysis for water treatment, but also for fuel generation, green synthesis, biomass degradation, chemical conversion, smart surface coating, antimicrobial surfaces, and self-cleansing materials [68]. This wide application of TiO_2_ may be due to the properties that it presents, such as high photocatalytic activity, low cost, low toxicity, high stability to irradiation, low environmental impact, and the capacity to regenerate without considerably losing its activity [63,65,68,74,75,76,77]. TiO_2_ has also a strong resistance against acid and alkali solutions [74]. When photons that present an energy equal or higher than the TiO_2_ bandgap excite electrons from the valence band to the conduction band, the photocatalytic process happens. Consequently, the vacancies in the valance band can oxidize the water molecules or ^-^OH ions adsorbed onto TiO_2_ surface generating ^•^OH radicals, which are strong oxidants capable to degrade the organic pollutants [76]. However, there are other ways to generate these radicals. In the Fenton process, for instance, they are generated from a mixture of H_2_O_2_ and Fe^2+^ in an acidic medium. This process in homogeneous mode is one of the most used photo-assisted options for the removal of pharmaceuticals, including antibiotics, from wastewater [78,79]. In heterogeneous photo-Fenton, iron-based solid catalysts, such as Fe_2_O_3_, are used, which allows their facile removal from the treated wastewaters [74]. Even though the previously referred photocatalytic methods are widely used and well established, many new photocatalysts have been developed and applied for pharmaceuticals removal in the last years, including oxides (e.g., ZnO, WO_3_, V_2_O_5_), sulfides (e.g., CdS, ZnS), oxyhalides (e.g., BiOCl, BiOBr), or conducting polymers, such as graphitic carbon nitride [64,74,77]. Most of them have been aimed at the treatment of wastewater from sewage treatment plants (STPs), however this review will evaluate if and how the new options are being, or may be, included on the treatment of aquaculture wastewater.

Table 2 depicts some photocatalytic strategies for the removal of aquaculture antibiotics. As it may be seen, among the several systems, which will be discussed in the subsequent sub-sections, TiO_2_ based photocatalysts are those with the widest expression in literature.

### 4.1. TiO_2_-Based Photocatalysts 

**Figure 4:**
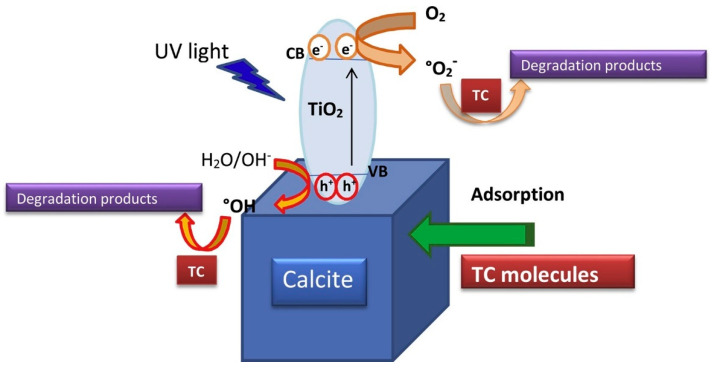
Representation of the photocatalytic process of calcite-titania-TC system. Reprinted from Belhouchet et al. [67] Copyright (2019), with permission from Elsevier.

A new efficient photocatalyst involving calcite and TiO_2_ was synthesized by Belhouchet et al. (2019) [67] using the sol-gel process in order to be applied on the degradation of tetracycline (TC), with the corresponding photocatalytic process been represented in Figure 4. 

The effect of different conditions, for instance, pH, TC initial concentration and calcite content, was studied. The best photodegradation rates for TC under UV light were observed for 1.5 g L^−1^ and 50 mg L^−1^ of catalyst and TC, respectively, and pH ≈ 7. A very interesting point of this study, which is not frequently assessed, was the evaluation of mineralization by measuring the total organic carbon (TOC). Even though mineralization did not occur in parallel and at the same extent as photodegradation, the employed photocatalyst was able to guarantee full mineralization of TC whatever the conditions. Authors stated that TC probably degraded into small intermediate metabolites of lower molecular weight than the original compound, which may explain the lack of parallelism between mineralization and photodegradation. 

Gaeta et al. (2020) [76] used an environmentally friendly procedure by a non-covalent one-pot approach to functionalize TiO_2_ with different porphyrins (Sn(IV) 5,10,15,20-tetrakis(4-pyridyl)porphyrin (SnT4), H_2_TCPP, and its Cu(II) and Zn(II) derivatives (CuTCPP and ZnTCPP, respectively)). TiO_2_ and the obtained porphyrin@TiO_2_ nanocomposites were tested as photocatalyst for the removal of OXA and OTC. Under simulated solar irradiation, OTC was more efficiently photodegraded (>30% after 10 min) than OXA (<10% after 10 min). This was related with the UV-vis spectra of the antibiotics—Whilst OTC absorbs in a large region of the visible light, OXA absorbs mostly in the UV and blue region of the solar spectrum. The introduction of TiO_2_ resulted in a much faster OXA photodegradation compared with that without it. The produced porphyrin@TiO_2_ nanocomposites were less efficient than TiO_2_ but also accelerated OXA photodegradation. SnT4@TiO_2_ displayed the poorest photocatalytic activity, which was related to its scarce surface functionalization. However, the three other systems (H_2_TCPP@TiO_2_, CuTCPP@TiO_2_, ZnTCPP@TiO_2_) evidenced an enhancement of the photodegradation capacity, with CuTCPP@TiO_2_ providing the most efficient results. In the case of OTC, neither the introduction of TiO_2_ or the porphyrin@TiO_2_ nanocomposites increased photodegradation within the first 10 min of irradiation. However, degradation rates progressively became divergent, and authors speculated that, after 40 min of irradiation, CuTCPP@TiO_2_ seemed more efficient that TiO_2_ alone.

Pereira et al. (2013) [65] constructed a pilot plant to investigate the TiO_2_-assisted solar-driven photodegradation of OXA and OTC. A concentration of 0.5 g L^−1^ and an initial pH of ≈7.5 were used to undergo kinetic studies and both single and binary solutions of the considered antibiotics were tested. Interestingly, the degradation profiles of each antibiotic were analogous either in single or binary solution. Less favorable kinetic rates were determined for OTC than for OXA in their individual solutions while both were removed nearly simultaneously from their binary solution. The use of TiO_2_ provided a 100% solar photocatalytic efficiency for both OXA and OTC and was also proved to allow for a high degree of mineralization (73 and 81%, respectively). Contrarily, mineralization was insignificant in the absence of the catalyst due to a simple transformation of antibiotics into more stable photoproducts. Moreover, Pereira et al. (2013) [65] also assessed the antibacterial activity and showed that the remaining organic compounds after photodegradation did not inhibit the growing of bacteria. Finally, Pereira et al. (2013) [65] observed that, among the inorganic ions which effect was tested, just the presence of PO_4_^3−^ reduced the TiO_2_ photocatalytic efficiency, while the rest (Cl^−^, SO_4_^2−^, NO_3_^−^, NH_4_^+^, and HCO_3_^−^) did not have substantial effects.

For the photocatalytic degradation of FLU an aqueous suspension of TiO_2_ assisted by simulated solar light was used by Palominos et al. (2008) [80]. Authors optimized the experimental conditions and concluded that the most important factor affecting photodegradation was pH (optimum value ≈ 6); on the contrary, the addition of hydrogen peroxide did not alter the efficiency of the process. Under optimized conditions, complete removal of FLU was very rapid (30 min). Mineralization was not exactly parallel to photodegradation, but also attained a good extent (80%) in a considered short time (60 min). Authors also evaluated the effect of the presence of scavengers and found that FLU photodegradation is marginally influenced by ^•^OH radicals, happening essentially through holes and the participation of the superoxide anions. A very interesting contribution of this work by Palominos et al. (2008) [80] was the study of the FLU solutions antibacterial activity after irradiation. An obvious correspondence between total FLU depletion and complete antibacterial activity inhibition was observed, evidencing the lack of biological activity of photoproducts from FLU photocatalysis, which was pointed as a valuable tool treatment to avoid antibiotics detrimental effects in the environment. These findings are in agreement with results obtained by Pereira et al. (2013) [65] for OXA and OTC.

In the study by Sirtori et al. (2009) [81], the degradation of FLU and nalidixic acid (NXA) in distilled water by a TiO_2_ photocatalytic process was evaluated at pilot scale. Photocatalysis with TiO_2_ brought similar results for both antibiotics, which were completely degraded within 25 min, although the mineralization of FLU was greater than that of NXA. At the end of FLU and NXA photocatalysis using solar radiation, the remaining dissolved organic carbon (DOC) was mainly attributed to carboxylic acids (48%) and to a mixture of propionic, acetic and oxalic acids (42%), respectively. Initially, FLU and NXA were shown to affect *Vibrio fischeri* with inhibitions of 80% and 70%, respectively, which decreased 55% and 35% after complete TiO_2_ assisted photodegradation.

**Table 2:** Photocatalysis of antibiotics used in aquaculture.

Reference	Antibiotics	Irradiation	Photocatalytic System	Conclusions
[67]	TC	UV	Calcite/TiO_2_	A content of 1.5 g L^−1^ of catalyst, 50 mg L^−1^ of TC, and pH ≈ 7 were the best conditions to effectively remove TC under UV light
[75]	CIP	Simulated sunlight	ZnSnO_3_	Co-precipitation showed better performance and stability in comparison with hydrothermal and template-assisted methods
				The prepared photocatalyst catalysed the degradation of CIP under simulated light irradiation and the antibacterial activity of CIP was largely decreased
[76]	OTC; OXA	Sunlight and UV	Porphyrin@TiO_2_	For OXA, the introduction of TiO_2_ as photocatalyst on the process caused a marked increase on photodegradation
				Under simulated solar irradiation (10 min), OTC photodegration was more than 30% larger than that of OXA
[82]	FLO	UV	UV/Na_2_S_2_O_8_	Rate constant increased linearly with increased PS concentration
				Presence of anions adversely affected FLO degradation performance
[83]	OTC	Visible light (20 W fluorescent lights)	UV/ZnO and UV/CuO/ZnO	UV/CuO/ZnO system was found to be more efficient than UV/ZnO
				The 90% of OTC degradation was accomplished for 10:1 molar ratio of Zn^2+^/Cu^2+^ and 0.4 g L^–1^ Nanocomposite
[84]	SMM	UV	UV/Zeolite/TiO_2_	Co-existent substances present in the aquaculture wastewater inhibited the photocatalytic decomposition of SMM
[80]	FLU	Simulated sunlight (500 W m^−2^ irradiance)	Simulated solar light/TiO_2_	The most important variable for FLU photodegradation was pH (optimal value ca. 6)
				FLU was completely eliminated within 30 min of irradiation
				The 80% of mineralization was accomplished after 60 min of irradiation
[65]	OXA; OTC	Simulated sunlight (1700 W Xe lamp)	Solar light/TiO_2_	Both single and mixture antibiotics’ solutions were used
				After complete removal of the antibiotics, photoproducts did not show antibacterial activity
[81]	FLU; NXA	Sunlight	TiO_2_	Photo-Fenton was more efficient than TiO_2_ in the degradation of NXA
			Photo-Fenton	A high degree of mineralisation was achieved in short irradiation times
[85]	THI	UV	UV/H_2_O_2_	Photodegradation rate decreased with the increase of the initial THI concentration
			UV/persulfate	Photodegradation rate increased with increasing oxidant dosage
[86]	SMX	High pressure Xe short arc lamp	Photo-assisted	Coupling nanofiltration with photocatalysts allowed for 80% of SMX removal
			nanofiltration	The membrane/photocatalyst system showed a good regeneration capacity
[87]	FLU; OXA;	UV-A	TiO_2_	Degradation of the quinolones was studied in mono-, binary and ternary compound systems
	NXA			Mono-compound systems showed the largest photocatalytic rates
[88]	CHL	Low-pressure mercury lamp	UV/H_2_O_2_	Antibacterial activity was absent after treatment, even at higher antibiotic initial concentrations than those commonly found in wastewaters

CHL—Chloramphenicol; CIP—Ciprofloxacin; DOM—Dissolved organic matter; ^3^DOM^*^—Triplet excited-state of DOM; ENR—Enrofloxacin; FLO—Florfenicol; FLU—Flumequine; NOR—Norfloxacin; NXA—Nalidixic acid; OMP—Ormetoprim; OTC—Oxytetracycline; OXA—Oxolinic acid; PA—Pipidemic acid; PS—Persulfate; PBS—Phosphate buffer solution; RS—Rosoxacin; SDZ—Sulfadiazine; SMM—Sulfamonomethoxine; *t*_1/2_—Half-life time; TC—Tetracycline; THI—Thiamphenicol; TRIM—Trimethoprim.

**Figure 5:**
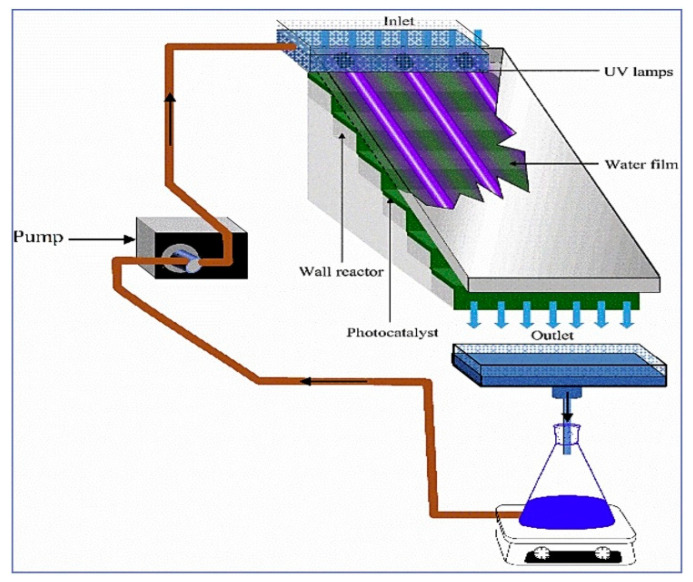
Representation of the pilot scale system composed by pump, UV lamp and photocatalyst. The dark arrow denotes the water circulation. Reprinted from Zeghioud et al. [87] Copyright (2019), with permission from Elsevier.

Zeghioud et al. (2019) [87] presented an important study at a pilot scale, which representation is presented in Figure 5, based on the TiO_2_ photocatalytic degradation of three quinolones (FLU, OXA and NXA) using their single, binary, and ternary solutions. 

The pilot scale system used UV irradiation and consisted of an original closed loop photoreactor with a supported photocatalyst consisting of TiO_2_ impregnated on cellulosic paper. Within 240 min, a complete degradation was reached for the three antibiotics. However, FLU was observed to reach all the evaluated degradation levels faster than the other two antibiotics. For instance, after 30 min, OXA and NXA reached 18 and 25% of degradation, respectively, while FLU degradation efficiency was already on its *t*_1/2_ (52%); after 150 min, when FLU was almost completely degraded (~92%), OXA and NXA were 62 and 78%, respectively. Related with mineralization, authors observed a progressive reduction of TOC over time, indicating antibiotics mineralization. OXA was the antibiotic with a faster mineralization and, after 6 h on the supported TiO_2_ under irradiation, its mineralization yield was above 65%, while FLU and NXA reached 57 and 43%, respectively. As compared with mono-compound systems, the binary and ternary systems displayed slower degradation rates. This was related to the competition between antibiotics and also their by-products for the available active sites on the photocatalyst surface, which was already reported by Sirtori et al. (2009) [81]. Competition was also referred as an inhibitory factor by Nomura et al. (2017) [84]. In their work, removal efficiency of sulfamonomethoxine (SMM) and its degradation intermediates formed by using photocatalysis by zeolite/TiO_2_ composites was investigated in fresh aquaculture wastewater. Coexistent substances in the wastewater were thought to inhibit the photocatalytic decomposition of SMM. However, this effect was present when TiO_2_ alone was used, but it was mitigated when using the composites. The higher efficiency of the composites was demonstrated by accomplishing a complete SMM removal within 30 min. Still, it has to be highlighted that the authors took into account the removal ascribed not only to photodegradation but also to adsorption. Later on, the same authors conducted a very similar study—in what concerns the composite composition, antibiotic and water matrix—but using a rotating advanced oxidation contactor and a composite sheet [89] instead of the powder composite used in the previous work [84]. The applied system efficiently removed SMM but, as before [84], the combination of adsorption and photocatalysis was considered key for attaining such efficiency.

TiO_2_ nanotube arrays and TiO_2_ nanowires on nanotube arrays were used by Do et al. (2019) [90] in the degradation of amoxicillin (AMX), ampicillin (AMP), doxycycline (DXC), OTC, lincomycin (LCM), VCM, sulfamethazine (SMZ), and SMX under UV-vis light. The nanomaterials allowed for a fast and efficient degradation of the eight antibiotics, which were completely removed after 20 min of treatment. 

Zhang et al. (2018) [86] applied an innovative strategy of photo-assisted nanofiltration for the treatment of aquaculture water based on the synergistic action of photocatalysis and nanofiltration membranes. For that purpose, authors built a multifunctional membrane assembled with g-C_3_N_4_, TiO_2_, carbon nanotubes (CNTs) and graphene oxide (GO), in which CNTs expanded the interlayer space between g-C_3_N_4_ sheets and enhanced GO stability and strength. Results on SMX revealed that nearly 18% was retained during the membrane filtration process without irradiation but about 80% of removal was obtained benefiting from the synergistic effect of photocatalysis and nanofiltration by the GO/CN/TiO-CNT membrane. Furthermore, authors proved the efficient mineralization of SMX during treatment since TOC removal, which was about 18% by the membrane without irradiation, was above 70% by the photo-assisted GO/CN/TiO-CNT membrane. 

Another interesting approach is the application of waste-based magnetic biochar (BC)/TiO_2_ composite materials on the photodegradation of SDZ and OXA [91]. SDZ *t*_1/2_ steeply decreased 3.9 and 3.4 times in presence of the magnetized BC functionalized with TiO_2_ and the BC functionalized with TiO_2_ and afterwards magnetized by an ex-situ approach, respectively. For OXA, the decrease was not that sharp but the photocatalysts permitted a decrease of 2.6 and 1.7 times in *t*_1/2_ for the referred materials, respectively. Additionally, mineralization rate for OXA was much slower than for SDZ and only possible in presence of the photocatalysts.

As mentioned before, TiO_2_ is among the most widely used photocalysts, not only due to its high photocatalytic activity but also to characteristics such as stability, low-cost, non-toxicity and corrosion resistance. However, most of published studies on water treatment using TiO_2_ have been carried out at laboratory scale, which is due to technical barriers that limit its practical application. First, the wide bandgap of TiO_2_ makes necessary UV irradiation for photocatalytic activation, which limits its use under sunlight. Finding strategies to shift the TiO_2_ optical response to the visible spectral range is essential to favor the widespread of its utilization in the photocatalytic removal of antibiotics from aquaculture wastewater. On the other hand, the low adsorption capacity to hydrophobic contaminants, high aggregation, and difficulties in the separation and recovery are important limitations for TiO_2_ practical application. Research on the development of novel and varied composites to overpass these limitations is advancing very fast and it is foreseeable that will help to extend the use of TiO_2_-based materials for the photocatalytic removal of antibiotics at real scale.

### 4.2. Other Photocatalysts 

Besides TiO_2_, many other photocatalysts have been prepared and used for the degradation of antibiotics from water. Degradation of FLO was photocatalyzed using UV irradiation in conjugation with sodium persulfate by Gao et al. (2015) [82]. This process shown to be more efficient than UV conjugated with H_2_O_2_ in what concerns both the photodegradation and the mineralization. Additionally, the concentration of sodium persulfate was shown to interfere with the FLO photodegradation rate, with the pseudo-first-order rate constant increasing linearly with increasing persulfate concentration. The effect of the presence of other species, namely the anions NO_3_^−^, Cl^−^, and HCO_3_^−^, ferrous ion, and HA, was also studied. It was concluded that the presence of the ferrous ion enhanced the FLO photodegradation; on the contrary, all the anions that tested unfavorably influenced the FLO degradation, as well as the presence of HA, which substantially decreased the degradation by radical scavenging and light-screening effect. As for the application of the process in real water samples, FLO degradation rate constants were lower than in deionized water. This may be due to the presence of constituents of the environmental water matrices, as scavenging ions or competing compounds. As for FLO mineralization, Gao et al. (2015) [82] found that even though this antibiotic was almost completely photodegraded after 1 h treatment, the decrease in TOC was only ~22%. A low mineralization compared with degradation has also been observed for other systems, for example, as above referred, for TC photocatalysis using calcite/TiO_2_ [67]. Differently, other authors have found that mineralization takes place in parallel with photodegradation, which was the case of the already referred work by Nomura et al. (2017) [84], who observed the complete mineralization of SMM when this was completely photodegraded by using zeolite/TiO_2_ composites.

Liu et al. (2019) [83] evaluated two systems (UV/ZnO and UV/CuO/ZnO; the latter being more efficient) for the degradation of OTC. The photocatalytic degradation rate achieved a maximum for a 10:1 Zn^2+^/Cu^2+^ molar ratio and 0.4 g L^–1^ nanocomposite concentration. Using these conditions, OTC photodegraded in about 90%. Comparing the systems, UV/CuO/ZnO was concluded to be more efficient than UV/ZnO, which was attributed to the doping of CuO, which effectively separated the photogenerated electron–hole pairs, promoted the photogenerated electrons transmission, slowed down the photoelectron–hole pair recombination, and generated more hydroxyl radicals for the degradation of OTC. Besides the molar ratio and the composite concentration, authors also studied other influencing factors as the calcination temperature, the irradiation time and the initial concentration of OTC. The most efficient results were obtained for 400 °C of calcination temperature, 3.5 h of irradiation time and 0.01 g L^–1^ of OTC initial concentration. Even though, amongst the considered factors, OTC initial concentration was the one with the largest influence on the photocatalytic rate, which drastically decreased with the increase of OTC initial concentration. This was associated to the covering of the CuO/ZnO photocatalyst surface with OTC, so restricting irradiation transmission and hampering the generation of electron–hole pairs.

As for THI, Wang et al. (2017) [85] investigated its degradation under UV radiation alone, comparing it with UV/H_2_O_2_ and UV/persulfate, under different conditions. First of all, it was concluded that UV/H_2_O_2_ and UV/persulfate considerably improved the degradation of THI compared to UV. Authors studied the effect of several parameters (e.g., initial THI concentration, oxidant dosage, pH, anions, and HA) on the THI degradation and found that the increase in the THI initial concentration decreased the photodegradation rate, which, however, increased with increasing oxidant dosage. As for the pH, neutral and acidic values favored the UV/H_2_O_2_ process, while the UV/persulfate process was more favorable under acidic and basic media. THI degradation was also affected by the presence of some ions, since both UV/H_2_O_2_ and UV/persulfate processes were markedly inhibited in presence of HCO^3−^; also, UV/H_2_O_2_ process presented a low inhibition in presence of NO^3−^ while Cl^−^ considerably improved the degradation accomplished with the UV/persulfate system. Regarding HA, they markedly inhibited the photocatalytic process for both systems, being the inhibitory effect more pronounced with the increase of HA concentration in the studied range (0–50 mg L^−1^). Wang et al. (2017) [85] also found that, after 120 min under irradiation, when THI was completely degraded, only a part of it was mineralized with UV/H_2_O_2_ process (DOC decreased just by 40%, pointing to the probable presence of organic intermediates), while for the UV/persulfate system it was mostly mineralized (DOC decreased in 92%). Apart from these differences between the studied systems, it is noteworthy to refer that mineralization and photodegradation did not occur in parallel for none of them, for example, after 10 min of irradiation, THI was 50% degraded but DOC persisted almost unaltered (97–98%) for both the UV/H_2_O_2_ and the UV/persulfate processes.

## 5. Future Challenges: A Critical Approach

More research is needed for a better understanding of the relationship between the antibiotics’ presence in the environment and the development of bacterial resistance and consequent effects on public health. However, it is clear that the growing antibiotic consumption, including veterinary consumption by aquacultures, is directly related with the environmental contamination with antibiotics. Therefore, considering the emergence of AMR, aquaculture needs to accomplish sustainable and environmentally friendly aquaculture practices to hamper its dispersion. Such practices should start by a prudent use of antibiotics, encouraging the use of alternatives, such as probiotics and essential oils, to promote fish health maintenance and disease prevention [5]. However, this change in the paradigm seems to be difficult and not to be happening in the near future. Therefore, since antibiotics are, and possibly will be, used in aquacultures for a long time, it is actually essential to research sustainable techniques for their removal from water, and here falls the importance of photolysis and photocatalysis. Even though the above-revised literature shows the impressive development of this field of research, there are some challenges that need to be addressed:

- Studies on antibiotics remediation by photodegradation are less than they should be—it is still needed to enlarge the scope of this area of investigation;
- In the specific case of the studies on antibiotics used in aquaculture, most of them approach the problem and investigate possible solutions but do not present a real application in aquaculture waters;
- Real environments are much more complex than laboratory-controlled conditions, so it would be necessary to develop computational models to predict the aquatic photochemical behavior of aquaculture antibiotics, as well as their different dissociation and/or metal complexation forms under different conditions;
- Further work on antibiotics’ mixtures should be carried out since they are mostly present simultaneously in real matrices and it may be anticipated that individual and simultaneous presence may represent a different behavior;
- More studies are also needed in what respects mineralization since it is crucial to understand if the photolysis/photocatalysis treatments offer the desired mineralization of the compound into CO_2_ and H_2_O;
- In the same line of thinking, antibacterial activity tests should be carried out to undoubtedly determine if, in the case of incomplete mineralization, photoproducts retain the activity of the parent compound—this is vital to consider if a treatment is effective;
- Another shortage in literature is the lack of studies in continuous operation mode and of upscaling studies as opposed to batch discontinuous laboratory studies—water treatments are usually implemented under continuous feeding, and it is important to scale-up the studies so as to understand if they present potential to be applied in a real situation.

## 6. Conclusions

Reviewed literature in this manuscript made evident that the study of aquaculture antibiotics’ photolysis and photocatalysis is an emerging field of research, with most of works having been published in the last decade. It has been shown that photodegradation may largely affect the persistence of antibiotics in the aquatic environment. Still, such photodegradation is dramatically affected by environmental factors such as the type of light, presence of ions, DOM, ROS, temperature, pH, salinity, type of matrix, or antibiotic initial concentration. Moreover, it has been observed that the effect of some of these factors may vary depending on the antibiotic (for example, DOM, salinity, pH, and type of matrix may either favor or hinder photodegradation) while other factors have been observed to have a consistent effect (for example, increasing antibiotic initial concentration hinders photodegradation due to competition for photons). Among the studied factors, DOM was shown to be especially influencing, where its net effect is the balance of two opposite actions, namely slowing down (by quenching ROS or due to inner filter effect) or increasing (by photosensitizing effect) the photodegradation rate. As for water treatment, photocatalysis has been shown to be an efficient treatment for the removal of aquaculture antibiotics. For this purpose, most of the tested photocatalysts are TiO_2_-based, with some novel and innovative strategies having been proposed. Results published up to date indicate that treatments drawn upon photodegradation are promising for a sustainable removal of antibiotics from aquaculture wastewater. Concern about the consequences of antibiotics presence in the environment, especially about AMR, is probably underneath the recent but impressive development of research in this area. However, much is yet to be done in view of the implementation of photodegradation-based treatments, the main challenges being their study under real conditions (antibiotics concentrations, real matrices, up-scaled experiments) and the full assessment of the treatment efficiency (mineralization, photoproducts’ identification and effluents’ antibacterial activity).

## References

[1] Gunning D., Maguire J., Burnell G. (2016). The Development of Sustainable Saltwater-Based Food Production Systems: A Review of Established and Novel Concepts. Water.

[2] Dar A.H., Rashid N., Majid I., Hussain S., Dar M.A. (2020). Nanotechnology interventions in aqua-culture and seafood preservation. Crit. Rev. Food Sci. Nutr..

[3] Zhao Y., Yang Q.E., Zhou X., Wang F.-H., Muurinen J., Virta M.P., Brandt K.K., Zhu Y.-G. (2020). Antibiotic resistome in the livestock and aquaculture industries: Status and solutions. Crit. Rev. Environ. Sci. Technol..

[4] Botta R., Asche F., Borsum J.S., Camp E.V. (2020). A review of global oyster aquaculture production and consumption. Mar. Policy.

[5] Romero J., Feijoó C.G., Navarrete P. Antibiotics in Aquaculture—Use, Abuse and Alternatives. InTechOpen. https://www.intechopen.com/books/health-and-environment-in-aquaculture/antibiotics-in-aquaculture-use-abuse-and-alternatives.

[6] FAO (2015). http://www.fao.org/fishery/regional-aquaculture-reviews/reviews-2015/en.

[7] Ciji A., Akhtar M.S. (2020). Nitrite implications and its management strategies in aquaculture: A review. Rev. Aquac..

[8] Lulijwa R., Rupia E.J., Alfaro A.C. (2020). Antibiotic use in aquaculture, policies and regulation, health and environmental risks: A review of the top 15 major producers. Rev. Aquac..

[9] Caruso G. (2016). Antibiotic Resistance in Fish Farming Environments: A Global Concern. J. Fish. Sci..

[10] Lounas R., Kasmi H., Chernai S., Amarni N., Ghebriout L., Meslem-Haoui N., Hamdi B., Thalassas A. (2020). Towards Sustainable Mariculture: Some Global Trends. Int. J. Mar. Sci..

[11] Heuer O.E., Kruse H., Grave K., Collignon P., Karunasagar I., Angulo F.J. (2009). Human Health Consequences of Use of Antimicrobial Agents in Aquaculture. Clin. Infect. Dis..

[12] WHO Global Action Plan on AMR. 2015. https://www.who.int/antimicrobial-resistance/global-action-plan/en/.

[13] Calisto V., Domingues M.R.M., Esteves V.I. (2011). Photodegradation of psychiatric pharmaceuticals in aquatic environments: Kinetics and photodegradation products. Water Res..

[14] Chowdhury R.R., Charpentier P.A., Ray M.B. (2011). Photodegradation of 17β-estradiol in aquatic solution under solar irradiation: Kinetics and influencing water parameters. J. Photochem. Photobiol. A Chem..

[15] Giang C.N.D., Sebesvari1 Z., Renaud F., Rosendahl I., Minh Q.H., Amelung W. (2015). Occurrence and Dissipation of the Antibiotics Sulfamethoxazole, Sulfadiazine, Trimethoprim, and Enrofloxacin in the Mekong Delta, Vietnam. PLoS ONE.

[16] Lam M.W., Tantuco K., Mabury S.A. (2003). PhotoFate: A new approach in accounting for the contribution of indirect photolysis of pesticides and pharmaceuticals in surface waters. Environ. Sci. Technol..

[17] Peuravuori J., Pihlaja K. (2009). Phototransformations of selected pharmaceuticals under low-energy UVA–vis and powerful UVB–UVA irradiations in aqueous solutions-the role of natural dis-solved organic chromophoric material. Anal. Bioanal. Chem..

[18] Lin A.Y.-C., Reinhard M. (2005). Photodegradation of common environmental pharmaceuticals and estrogens in river water. Environ. Toxicol. Chem..

[19] Young R.B., Latch D.E., Mawhinney D.B., Nguyen T.-H., Davis J.C.C., Borch T. (2013). Direct photodegradation of androstenedione and testosterone in natural sunlight: Inhibition by dis-solved organic matter and reduction of endocrine disrupting potential. Environ. Sci. Technol..

[20] Carlos L., Mártire D.O., Gonzalez M.C., Gomis J., Bernabeu A., Amat A.M., Arques A. (2012). Photochemical fate of a mixture of emerging pollutants in the presence of humic substances. Water Res..

[21] Faust B.C., Zepp R.G. (1993). Photochemistry of aqueous iron (III)-polycarboxylate complexes: Roles in the chemistry of atmospheric and surface waters. Environ. Sci. Technol..

[22] Wu F., Deng N. (2000). Photochemistry of hydrolytic iron (III) species and photoinduced degradation of organic compounds. A minireview. Chemosphere.

[23] Pozdnyakov I., Sherin P., Bazhin N., Plyusnin V. (2018). [Fe(Ox)_3_]^3−^ complex as a photodegradation agent at neutral pH: Advances and limitations. Chemosphere.

[24] Ge L., Chen J., Qiao X., Lin J., Cai X. (2009). Light-source-dependent effects of main constituents on photodegradation of phenicol antibiotics: Mechanism and kinetics. Environ. Sci. Technol..

[25] Lin J.-S., Pan H.-Y., Liu S.-M., Lai H.T. (2010). Effects of light and microbial activity on the degra-dation of two fluoroquinolone antibiotics in pond water and sediment. J. Environ. Sci. Health B.

[26] Sturini M., Speltini A., Maraschi F., Pretali L., Profumo A., Fasani E., Albini A., Migliavacca R., Nucleo E. (2012). Photodegradation of fluoroquinolones in surface water and antimicrobial activity of the photoproducts. Water Res..

[27] Guerard J.J., Chin Y.-P. (2012). Photodegradation of Ormetoprim in Aquaculture and Stream-Derived Dissolved Organic Matter. J. Agric. Food Chem..

[28] Lai H.-T., Lin J.-J. (2009). Degradation of oxolinic acid and flumequine in aquaculture pond waters and sediments. Chemosphere.

[29] Wei X., Chen J., Xie Q., Zhang S., Li Y., Zhang Y., Xie H. (2015). Photochemical behavior of anti-biotics impacted by complexation effects of concomitant metals: A case for ciprofloxacin and Cu(II). Environ. Sci. Process. Impacts.

[30] Pouliquen H., Delépée R., Larhantec-Verdier M., Morvan M.-L., Bris H.L. (2007). Comparative hydrolysis and photolysis of four antibacterial agents (oxytetracycline, oxolinic acid, flumequine and florfenicol) in deionised water, freshwater and seawater under abiotic conditions. Aquaculture.

[31] Leal J.F., Esteves V.I., Santos E.B.H. (2016). Use of sunlight to degrade oxytetracycline in marine aquaculture’s waters. Environ. Pollut..

[32] Leal J.F., Esteves V.I., Santos E.B.H. (2019). Solar photodegradation of oxytetracycline in brackish aquaculture water: New insights about effects of Ca^2+^ and Mg^2+^. J. Photochem. Photobiol. A Chem..

[33] Liang C., Zhao H., Deng M., Quan X., Chen S., Wang H. (2015). Impact of dissolved organic matter on the photolysis of the ionizable antibiotic norfloxacin. J. Environ. Sci..

[34] Li K., Zhang P., Ge L., Ren H., Yu C., Chen X., Zhao Y. (2014). Concentration-dependent photo-degradation kinetics and hydroxyl-radical oxidation of phenicol antibiotics. Chemosphere.

[35] Louros V.L.D., Silva C.P., Nadais H., Esteves V.I., Lima D.L.D. (2020). Oxolinic acid in aquaculture waters: Can natural attenuation through photodegradation decrease its concentration?. Sci. Total. Environ..

[36] Oliveira C., Lima D.L.D., Silva C.P., Calisto V., Otero M., Esteves V.I. (2019). Photodegradation of sulfamethoxazole in environmental samples: The role of pH, organic matter and salinity. Sci. Total. Environ..

[37] Wang H., Yao H., Sun P., Li D., Huang C.-H. (2016). Transformation of Tetracycline Antibiotics and Fe(II) and Fe(III) Species Induced by Their Complexation. Environ. Sci. Technol..

[38] McNeill K., Canonica S. (2016). Triplet state dissolved organic matter in aquatic photochemistry: Reaction mechanisms, substrate scope, and photophysical properties. Environ. Sci. Process. Impacts.

[39] Sharpless C.M., Blough N.V. (2014). The importance of charge-transfer interactions in determining chromophoric dissolved organic matter (CDOM) optical and photochemical properties. Environ. Sci. Process. Impacts.

[40] Aguer J.P., Richard C., Andreux F. (1999). Effect of light on humic substances: Production of reactive species. Analusis.

[41] Silva C.P., Lima D.L.D., Otero M., Esteves V.I. (2016). Photosensitized Degradation of 17 β-estradiol and 17 α-ethinylestradiol: Role of Humic Substances Fractions. J. Environ. Qual..

[42] Silva C.P., Lima D.L.D., Groth M.B., Otero M., Esteves V.I. (2016). Effect of natural aquatic humic substances on the photodegradation of estrone. Chemosphere.

[43] Oliveira C., Lima D.L.D., Silva C.P., Otero M., Esteves V.I. (2016). Photodegradation behaviour of estriol: An insight on natural aquatic organic matter influence. Chemosphere.

[44] Louros V.L., Silva C.P., Nadais H., Esteves V.I., Lima D.L.D. (2020). Photodegradation of sulfadiazine in different aquatic environments—Evaluation of influencing factors. Environ. Res..

[45] Brezonik P.L., Fulkerson-Brekken J. (1998). Nitrate-Induced Photolysis in Natural Waters: Controls on Concentrations of Hydroxyl Radical Photo-Intermediates by Natural Scavenging Agents. Environ. Sci. Technol..

[46] Chen Y., Zhang K., Zuo Y. (2013). Direct and indirect photodegradation of estriol in the presence of humic acid, nitrate and iron complexes in water solutions. Sci. Total. Environ..

[47] Ge L., Na G., Zhang S., Li K., Zhang P., Ren H., Yao Z. (2015). New insights into the aquatic pho-tochemistry of fluoroquinolone antibiotics: Direct photodegradation, hydroxyl-radical oxidation, and antibacterial activity changes. Sci. Total. Environ..

[48] Cacciari D., Reynoso E., Spesia M.B., Criado S., Biasutti M.A. (2017). Vancomycin-sensitized pho-tooxidation in the presence of the natural pigment vitamin B2: Interaction with excited states and photogenerated ROS. Redox Rep..

[49] Ge L., Zhang P., Halsall C., Li Y., Chen C.-E., Li J., Sun H., Yao Z. (2019). The importance of reactive oxygen species on the aqueous phototransformation of sulfonamide antibiotics: Kinetics, pathways, and comparisons with direct photolysis. Water Res..

[50] Hao Z., Guo C., Lv J., Zhang Y., Zhang Y., Xu J. (2019). Kinetic and mechanistic study of sulfadimidine photodegradation under simulated sunlight irradiation. Environ. Sci. Eur..

[51] Chen Y., Hu C., Qu J., Yang M. (2008). Photodegradation of tetracycline and formation of reactive oxygen species in aqueous tetracycline solution under simulated sunlight irradiation. J. Photochem. Photobiol. A Chem..

[52] Leal J.F., Esteves V.I., Santos E.B.H. (2015). Does light-screening by humic substances completely explain their retardation effect on contaminants photo-degradation?. J. Environ. Chem. Eng..

[53] Li Y., Rashid A., Wang H., Hu A., Lin L., Yu C.-P., Chen M., Sun Q. (2018). Contribution of biotic and abiotic factors in the natural attenuation of sulfamethoxazole: A path analysis approach. Sci. Total. Environ..

[54] Doll T.E., Frimmel F.H. (2003). Fate of pharmaceuticals-photodegradation by simulated solar UV-light. Chemosphere.

[55] Jürgens M.D., Holthaus K.I.E., Johnson A.C., Smith J.J.L., Hetheridge M., Williams R.J. (2002). The potential for estradiol and ethinylestradiol degradation in English rivers. Environ. Toxicol. Chem..

[56] Liu B., Wu F., Deng N.S. (2003). UV-light induced photodegradation of 17α-ethynylestradiol in aqueous solutions. J. Hazard. Mater..

[57] Liu B., Liu X.L. (2004). Direct photolysis of estrogens in aqueous solutions. Sci. Total. Environ..

[58] Neamtu M., Frimmel F.H. (2006). Photodegradation of endocrine disrupting chemical nonylphenol by simulated solar UV-irradiation. Sci. Total. Environ..

[59] An Y., De Ridder D.J., Zhao C., Schoutteten K., Vanden B., Zheng H., Chen G., Vanhaecke L. (2016). Adsorption and photocatalytic degradation of pharmaceuticals and pesticides by carbon doped-TiO_2_ coated on zeolites under solar light irradiation. Water Sci. Technol..

[60] Cuervo L., Wielens B.R., Salmoria A.D., Dallegrave A., Ost F., Lavayen V.T., Sirtori C. (2019). Degradation of pharmaceuticals in different water matrices by a solar homo/heterogeneous photo-Fenton process over modified alginate spheres. Environ. Sci. Pollut. Res..

[61] De la Cruz R.F., Dantas J., Giménez S., Esplugas N. (2013). Photolysis and TiO_2_ photocatalysis of the pharmaceutical propranolol: Solar and artificial light. Appl. Catal. B Environ..

[62] Perini J.A.L., Tonetti A.L., Vidal C., Montagner C.C., Nogueira R.F.P. (2018). Simultaneous degra-dation of ciprofloxacin, amoxicillin, sulfathiazole and sulfamethazine, and disinfection of hospital effluent after biological treatment via photo-Fenton process under ultraviolet germicidal irradiation. Appl. Catal. B Environ..

[63] Tzeng T.-W., Wang S.-L., Chen C.-C., Tan C.-C., Liu Y.-T., Chen T.-Y., Tzou Y.-M., Chene C.C., Hung J.T. (2016). Photolysis and photocatalytic decomposition of sulfamethazine antibiotics in an aqueous solution with TiO_2_. RSC Adv..

[64] Yang X., Chen Z., Zhao W., Liu C., Qian X., Zhang M., Wei G., Khan F., Ng Y.H., Ok Y.S. (2021). Recent advances in photodegradation of antibiotic residues in water. Chem. Eng. J..

[65] Pereira J.H.O.S., Reis A.C., Queirós D., Nunes O.C., Borges M.T., Vilar V.J.P., Boaventura R.A.R. (2013). Insights into solar TiO2-assisted photocatalytic oxidation of two antibiotics employed in aquatic animal production, oxolinic acid and oxytetracycline. Sci. Total. Environ..

[66] Ameta R., Solanki M.S., Benjamin S., Ameta S.C. (2018). Photocatalysis in Advanced Oxidation Processes for Wastewater Treatment: Emerging Green Chemical Technology. Photocatalysis.

[67] Belhouchet N., Hamdia B., Chenchouni H., Bessekhouad Y. (2019). Photocatalytic degradation of tetracycline antibiotic using new calcite/titania nanocomposites. J. Photochem. Photobiol. A Chem..

[68] Fontana K.B., Lenzi G.G., Seára E.C.R., Chaves E.S. (2018). Comparison of photocatalysis and pho-tolysis processes for arsenic oxidation in water. Ecotoxicol. Environ. Saf..

[69] Velempini T., Pillay P.K. (2021). Recent developments in the use of metal oxides for photocatalytic degradation of pharmaceutical pollutants—A review. Mater. Today Chem..

[70] Babić S., Ćurković L., Ljubas D., Ćizmić M. (2017). TiO_2_ assisted photocatalytic degradation of macrolide antibiotics. Curr. Opin. Green Sustain. Chem..

[71] Bagheri S., Termehyousefi A., Do T.O. (2017). Photocatalytic pathway toward degradation of environmental pharmaceutical pollutants: Structure, kinetics and mechanism approach. Catal. Sci. Technol..

[72] Boxi S.S., Paria S. (2015). Visible light induced enhanced photocatalytic degradation of organic pollutants in aqueous media using Ag doped hollow TiO_2_ nanospheres. RSC Adv..

[73] Wang P., Zhou T., Wang R., Lim T.-T. (2011). Carbon-sensitized and nitrogen-doped TiO_2_ for photocatalytic degradation of sulfanilamide under visible-light irradiation. Water Res..

[74] Antonopoulou M., Kosma C., Albanis T., Konstantinou I. (2021). An overview of homogeneous and heterogeneous photocatalysis applications for the removal of pharmaceutical compounds from real or synthetic hospital wastewaters under lab or pilot scale. Sci. Total. Environ..

[75] Dong S., Cui L., Zhang W., Xia L., Zhou S., Russell C.K., Fand M., Feng J., Sun J. (2020). Double-shelled ZnSnO_3_ hollow cubes for efficient photocatalytic degradation of antibiotic wastewater. Chem. Eng. J..

[76] Gaeta M., Sanfilippo G., Fraix A., Sortino G., Barcellona M., Conti G.O., Fragalà M.E., Ferrante M., Purrello R., D’Urso A. (2020). Photodegradation of Antibiotics by Noncovalent Porphyrin-Functionalized TiO_2_ in Water for the Bacterial Antibiotic Resistance Risk Management. Int. J. Mol. Sci..

[77] Kutuzova A., Dontsova T., Kwapinski W. (2021). Application of TiO2-Based Photocatalysts to Antibiotics Degradation: Cases of Sulfamethoxazole, Trimethoprim and Ciprofloxacin. Catalysts.

[78] Koltsakidou A., Antonopoulou M., Εvgenidou Ε., Konstantinou I., Lambropoulou D. (2019). A comparative study on the photo-catalytic degradation of Cytarabine anticancer drug under Fe^3+^/H_2_O_2_, Fe^3+^/S_2_O_8_^2−^, and [Fe(C_2_O_4_)_3_]_3−_/H_2_O_2_ processes. Kinetics, identification, and in silico toxicity assessment of generated transformation products. Environ. Sci. Pollut. Res..

[79] Mirzaei A., Chen Z., Haghighat F., Yerushalmi L. (2017). Removal of pharmaceuticals from water by homo/heterogonous Fenton-type processes—A review. Chemosphere.

[80] Palominos R., Freer J., Mondaca M.A., Mansilla H.D. (2008). Evidence for hole participation during the photocatalytic oxidation of the antibiotic flumequine. J. Photochem. Photobiol. A Chem..

[81] Sirtori C., Zapata A., Malato S., Gernjak W., Fernández-Alba A.R., Aguera A. (2009). Solar photo-catalytic treatment of quinolones: Intermediates and toxicity evaluation. Photochem. Photobiol. Sci..

[82] Gao Y.-Q., Gao N.-Y., Deng Y., Yin D.-Q., Zhang Y.-S. (2015). Degradation of florfenicol in water by UV/Na_2_S_2_O_8_ process. Environ. Sci. Pollut. Res..

[83] Liu J., Yu X., Wang L., Guo M., Tian S., Zhu W. (2019). Photocatalytic degradation of oxytetracycline hydrochloride pollutants in marine aquaculture wastewater under visible light. J. Environ. Sci. Health Part A Toxic/Hazard. Subst. Environ. Eng..

[84] Nomura Y., Fukahori S., Fukada H., Fujiwara T. (2017). Removal behaviors of sulfamonomethoxine and its degradationintermediates in fresh aquaculture wastewater using zeolite/TiO_2_ composites. J. Hazard. Mater..

[85] Wang F., Wang W., Yuan S., Wang W., Hu Z.-H. (2017). Comparison of UV/H_2_O_2_ and UV/PS processes for the degradation of thiamphenicol in aqueous solution. J. Photochem. Photobiol. A Chem..

[86] Zhang Q., Chen S., Fan X., Zhang H., Yu H., Quan X. (2018). A multifunctional graphene-based nanofiltration membrane under photo-assistance for enhanced water treatment based on layer-by-layer sieving. Appl. Catal. B Environ..

[87] Zeghioud H., Kamagate M., Coulibaly L.S., Rtimi S., Assadi A.A. (2019). Photocatalytic degradation of binary and ternary mixtures of antibiotics: Reactive species investigation in pilot scale. Chem. Eng. Res. Des..

[88] Zuorro A., Fidaleo M., Fidaleo M., Lavecchia R. (2014). Degradation and antibiotic activity reduction of chloramphenicol in aqueous solution by UV/H2O2 process. J. Environ. Manag..

[89] Nomura Y., Fukahori S., Fujiwara T. (2020). Removal of sulfamonomethoxine and its transformation by products from fresh aquaculture wastewater by a rotating advanced oxidation contactor equipped with zeolite/TiO_2_ composite sheets. Process. Saf. Environ. Prot..

[90] Do T.C.M.V., Nguyen D.Q., Nguyen K.T., Le P.H. (2019). TiO_2_ and Au-TiO_2_ nanomaterials for rapid photocatalytic degradation of antibiotic residues in aquaculture wastewater. Materials.

[91] Silva C.P., Pereira D., Calisto V., Martins M.A., Otero M., Esteves V.E., Lima D.L.D. (2021). Biochar-TiO_2_ magnetic nanocomposites for photocatalytic solar-driven removal of antibiotics from aquaculture effluents. J. Environ. Manag..

